# How I do it: Radiofrequency ablation

**Published:** 2008-05

**Authors:** Roy Santosham, Bhawna Dev, Preeti Priyadarshini, Harsha Chadga, C Anupama, Rajiv Santosham, Rajan Santosham, S Vishnu

**Affiliations:** Department of Radiology, Sri Ramachandra University, Porur Chennai, India; 1 Santosham Chest Hospital, Egmore Chennai, India

**Keywords:** Radiofrequency ablation, technique

## Abstract

Over the past decade, image-guided tumor ablation using thermal energy has emerged as a promising technique for treating focal, primary or secondary, nonoperable tumors. Radiofrequency ablation (RFA) is minimally invasive and requires less resources, time, and recovery period and is, moreover, relatively inexpensive. RFA has been used to treat tumors located in the liver, lung, bone, kidneys, brain, thyroid, breast, and pancreas. This article will describe how to choose an appropriate case; precisely place the needle into the tumor; the precautions to be taken before, during, and after the procedure; probable complications; and the follow-up of patients undergoing ablation.

The various treatment options available to treat inoperable tumors are embolization; chemoembolization; chemotherapy, radiotherapy; tumor ablation by ethanol injection; ablation techniques like cryoablation; and ablation by heat-generating energies like microwave, laser, and focused ultrasound. RFA is also a heat-generating treatment option which, in recent years, has gained wide acceptance in the management of inoperable tumors.

The volume and shape of ablation achieved by ethanol cannot be predetermined, since ethanol is liquid and flows into areas of relatively less resistance, leading to inadequate ablation of some parts of the tumor and inappropriate ablation of normal areas. This makes ethanol unsuitable for treating tumors in relatively soft organs like the brain, lung, and the non-cirrhotic liver. The promise shown by ethanol injection in the treatment of small liver tumors has been dampened because multiple consecutive sessions are needed to successfully treat even small tumors and because of its ineffectiveness in the treatment of colorectal metastases.[1,2]

Of all the thermal ablation techniques mentioned above, cryoablation has been investigated the most.[3,4] Though the overall prognosis of patients undergoing cryoablation for liver tumors is comparable with that of surgery, in most cases laparotomy is still warranted. The time taken to treat large tumors by cryoablation is less as compared with RFA, but RFA has been reported to have lower recurrence rates (2.2% *vs* 13.6%) and complication rates (3.3% *vs* 40.7%).[5]

Heat-generating ablative techniques are very useful since the treated area can be precisely predetermined and also because the morbidity is minimal as the procedures are relatively noninvasive. They can be performed as day-care procedures. Though laser ablation has been claimed to be highly effective in the treatment of HCC and colorectal liver metastases by German investigators,[6] RFA is still the preferred technique.[7]

## Principle

Human cells undergo coagulative necrosis with temperatures above 50°C.[8,9] With increasing temperature, the time required for cells to undergo denaturation decreases. Permanent damage to the cell membrane occurs above 60°C and is followed by desiccation and charring.

During RFA, a high alternating current of approximately 480 KHz is passed into the tumor through a needle (electrode); this causes ionic agitation with resultant frictional heat production, finally leading to permanent cell death.[10] The amount of heat deposited in the tissue is proportional to the strength of the current and the resistance of the tissue and is inversely proportional to the fourth power of the distance from the tip of the needle.

Excessive heat deposition (>100°C), causes uncontrolled charring and gas formation leading to a break in the electrical circuit. A precise control over temperature, power, and time is required while performing RFA. In larger tumors, where more time is required to ablate the tumor completely, excessive charring is controlled by injection of saline into the tissue or by cooling the needle tip with chilled saline.[11],[12] Another limitation is the presence of large vessels immediately adjacent to the tumor. Flowing blood within these vessels carry away heat and prevent adequate heating of the tumor; the so-called ‘heat-sink’ effect.

## RFA: How I do it

The technique that we follow is described in the following text. The various steps include preoperative evaluation; approach planning, i.e., percutaneous, laparoscopy/thoracoscopy, or open surgery; anesthesia; needle placement; heating strategy; and follow up.[13,14]

## Preoperative Evaluation

The preoperative evaluation may differ depending upon the organ to be ablated, but certain basic principles are common for all organ systems. Evaluation of all tumors begins with a review of all the pertinent cross-sectional images. A note should be made of the size and number of tumors, anatomical relationship of the tumor to vital structures, and the operability and feasibility of safe needle access. If the tumor is operable, the option of surgery is always given to the patient. The size of the tumor should be less than 5 cm if a complete cure is the aim. For larger tumors, the main role of RFA is to debulk the lesion prior to chemotherapy or for pain relief.

A blood coagulation assessment consisting of a complete blood count, hemoglobin, hematocrit, prothrombin time, partial prothrombin time, and platelet count is mandatory.

## Organ-specific Evaluation

### Liver:

Potential candidates are those with less than five tumors, with each measuring less than 5 cm, and with no distant metastases. Tumors located within 5 cm of each other have poor results because of increased tumor bulk and the possibility of incomplete ablation.[13]

A baseline assessment of serum alpha fetoprotein in patients with hepatocellular carcinoma and serum carcinoembryonic antigen in those with colorectal metastases is done. Excessive primary tumor burden, untreatable extrahepatic metastases, Child's class C cirrhosis, and infection are all contraindications for RFA.[13]

### Lung:

We perform RFA only in those patients who are not candidates for surgery. This applies to primary and secondary tumors. A complete cure can be achieved in tumors less than 5 cm in size and located more than 1 cm from the hilum. Tumors close to the hilum often do not get completely ablated due to their proximity to large vessels, which prevent optimal heating due to the heat-sink effect. Electrodes, due to their large caliber, when inserted close to the hilar structures may cause life-threatening hemoptysis. For larger tumors, the aim is to debulk them to aid chemotherapy/radiotherapy and for palliation of symptoms like pain and dyspnea. Whenever we traverse aerated lung, we always have a thoracic surgeon standing by with an intercostal drainage tube to manage large pneumothoraces.

### Bone - osteoid osteoma:

The precise identification of the osteoid osteoma nidus on CT scan is a must prior to performing RFA. An MRI is often performed as a baseline study to document the extent of marrow edema, the regression of which helps document successful treatment.

## System Description

### RF generator [Figure 1]:

**Figure 1:**
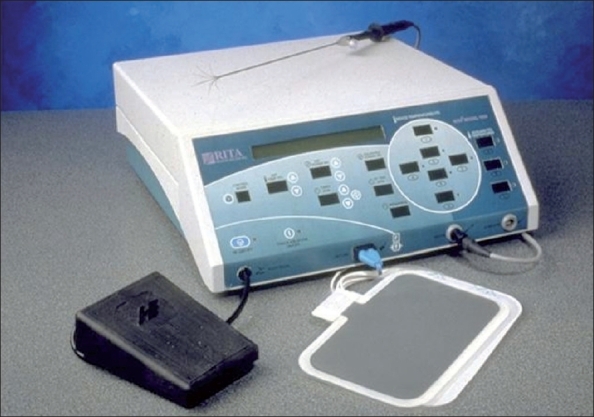
RITA Medical Systems Model 1500 X Electrosurgical Radi-ofrequency Generator

We use the Model 1500X Electrosurgical Radiofrequency Generator (Rita Medical Systems, USA). This provides monopolar radiofrequency and delivers 150 watts of RF power in most modes with up to 200 watts of RF power in the infusion mode.

### RF electrode:

**Figure 2(A-C):**
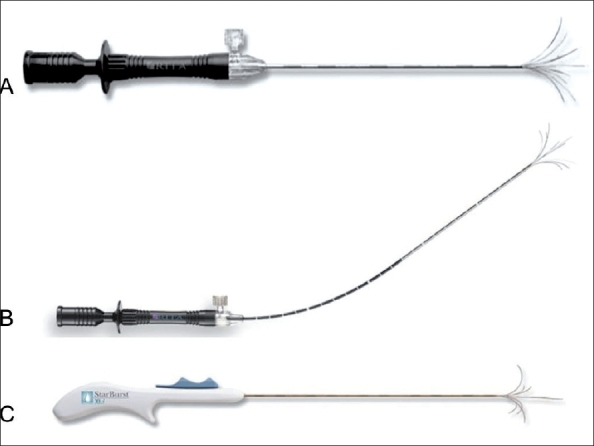
Different types of electrodes: (A) Star Burst, (B) Flexi, (C) Star Burst Xli

The electrode consists of deployable array electrodes (hooks). Some, or all, are equipped with thermocouples, depending upon the model. Electrodes are available in different lengths and designs [Figure 2].

### Main cable for device:

The cable connects and delivers RF energy from the generator to the electrode.

### Dispersive electrodes:

These complete the electrical circuit and provide the return path for the RF energy applied by the device.

## Needle Placement

The key to complete and successful ablation is the precise placement of the electrode within the tumor. The electrode may be introduced into tumors percutaneously under USG, CT scan, or MRI image guidance or by laparoscopic or open surgical means.

Before the needle is inserted, the point of entry, a safe trajectory, and the end position of the needle are planned. Once the needle has been inserted, the entry point is confirmed by placing a contrast-soaked cotton pledget over the lesion and taking a few axial sections. Then, if required, local anesthesia may be given along the tract up to the surface of the target organ. Subsequently, the RFA electrode is positioned along the anesthetized tract such that its tip lies approximately 1 cm proximal to the geometric center of the lesion. The expandable array of electrodes is opened out in stages and the tumor progressively ablated. Tumor ablation must be performed using sustained progressive heating. This is to ensure that desired temperatures are reached without unwanted carbonization (charring), which can hamper the flow of electricity and further heating.

**Figure 3:**
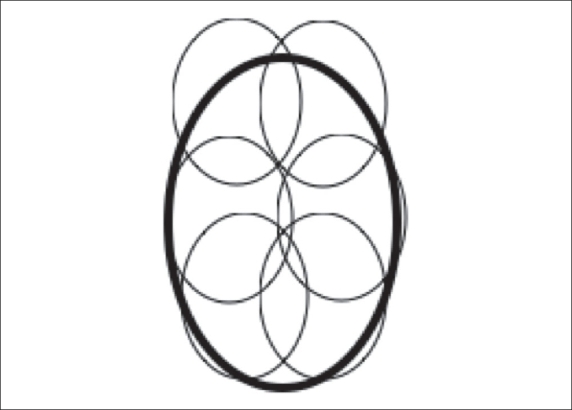
Line diagram showing the concept of overlapping ablations

If the aim of treatment is a cure, the entire tumor, along with a surrounding 1 cm cuff of normal tissue, should be ablated. To treat larger tumors, multiple overlapping ablations are required to build a composite thermal injury footprint of sufficient size to kill the entire tumor and to provide the desired tumor-free margin. For example, a near spherical tumor of 6 cm size would require eight overlapping ablations [Figure 3] with an electrode that would open 5 cm. Under USG guidance, it is preferable to ablate the deeper portions of the tumor prior to the superficial parts to prevent obscuration of the deeper parts by microbubbles produced during ablation of the superficial parts.

For bone tumors, an insulating guiding needle, probably of 13 G size or larger, which can be trephined or malleted precisely into the lesion, is required. Once in position, the electrode is coaxially passed through the guiding needle into the nidus and the ablation is performed.

## Heating Strategies [Figure 4]

**Figure 4:**
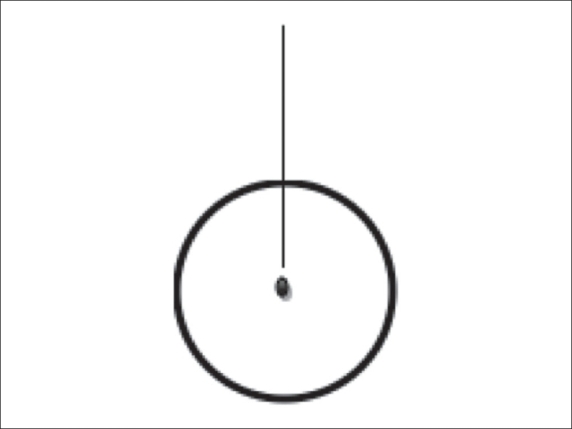
Line diagram showing the position of the electrode in a round tumor volume

Though the vendors have prescribed heating strategies for different organs, we have deviced our own. Our heating strategies for liver and lung tumors, for a 5-cm-sized ablation are shown in Tables 1 and 2. The electrode is deployed 1cm proximal to the target lesion for a 3-cm or 4-cm ablation and 1.5 cm proximal to the center for a 5-cm ablation [Figure 1].

**Table 1:** Heating strategy in the liver for a 5-cm ablation

Deployment (cm)	Target temp (degrees)	Power set (watts)	Timer (min)	Duration of ablation (min)
2	80	70	20	Till target temp achieved
	90	80	20	05
3	90	90	15	02
	100	100	13	05
4	100	110	05	03
5	110	120	05	05, Cool down

**Table 2:** Heating strategy in the lung for a 5-cm ablation

Deployment (cm)	Target temp (degrees)	Power set (watts)	Timer (min)	Duration of ablation (min)
2	70	60	20	Till target temp archived
3	80	70	20	02
	90	80	18	05
4	90	90	13	03
	90	100	10	05
5	110	120	05	05, Cool down

Osteoid osteoma ablation:

The nidus is heated at 90° for 6 min.

## Follow-up

**Figure 5(A, B):**
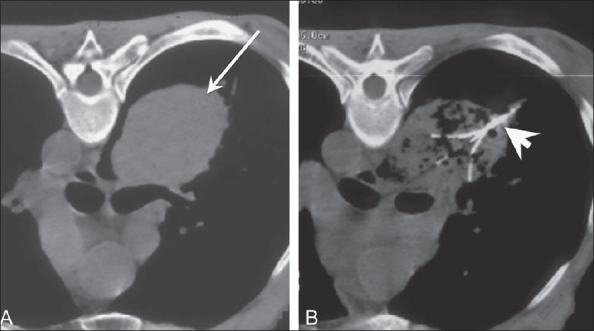
Bronchogenic carcinoma. Pre-ablation axial CT scan in the prone position (A) shows a large mass (arrow) in the lung. A similar image obtained during ablation (B) shows the electrode (arrowhead) in the mass

**Figure 6(A, B):**
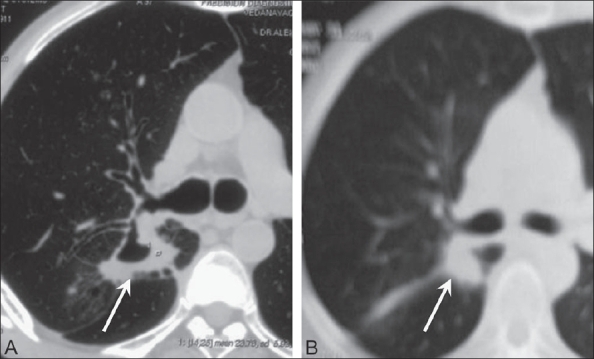
Bronchogenic carcinoma. Axial CT scan images obtained 3 months (A) and 9 months (B) following ablation of the same lung mass shown in Figure 5. The lesion shows significant reduction in size with cavitation (arrow) at 3 months and with further reduction (arrow) at 9 months
- b. Contrast enhancement: No central or peripheral enhancement is expected at any time after ablation [Figure 7].

We follow our cases at 1, 3, 6, and 18 months intervals. Three criteria are evaluated; these are:

- a. Size of the lesion: It is expected to increase in the first 1 week to 1 month due to reactive changes after ablation. At 3 months, the lesion should be equal to or smaller than the preprocedural size. Considerable reduction in the size is expected at 6 months and thereafter [Figures 5, 6].

**Figure 7(A-C):**
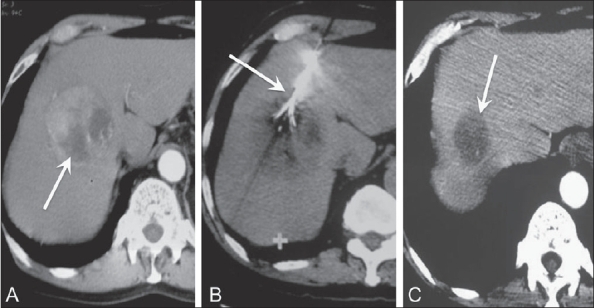
Hepatoma. The pre-ablation axial CT scan (A) shows a heterogeneous mass (arrow). The electrode is seen within the mass (arrow) during ablation (B). The mass shows significant reduction in size with absence of enhancement (arrow) 9 months (C) after the procedure
- c. Tumor markers: Elevation in organ-specific tumor markers points to recurrence or new lesions.

**Figure 8(A-C):**
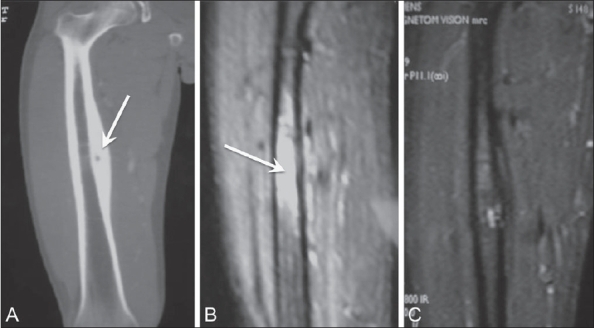
Osteoid osteoma. Pre-ablation CT scan (A) shows the nidus (arrow) of the osteoid osteoma; there is marrow edema (arrow) on a STIR MRI (B). The post-ablation MRI (C) shows complete absence of marrow edema

In patients with osteoid osteoma, pain relief is the best way to assess the success of the treatment; we also perform MRI to document the reduction in bone marrow edema around the nidus [Figure 8].

## Complications

The commonest complication encountered is the ‘postablation syndrome,’ which presents with constitutional symptoms like fever and arthralgia. Other expected complications are thermal injury, pleural effusion, and pneumothorax. Three of our lung tumor patients developed pulmonary edema, which was recognized in time and the procedure was abandoned. One patient had negligible pneumothorax. Another patient who was treated for osteoid osteoma had minor skin burns.

Other potential complications are arrhythmias, hemoptysis, hemothorax, abscess formation, formation of bronchopleural fistula, bleeding, and necrosis of adjacent organs.

## Conclusion

In the recent years RFA has definitely become a preferred mode of treatment for unresectable tumors of liver, lungs, and bone. This minimally invasive percutaneous technique is widely accepted by surgeons also as it offers a better prognosis than leaving the tumor alone. It is one of the best methods to achieve local tumor control. In larger tumors, RFA is more effective when combined with chemotherapy and arterial embolization. It is easily available, better tolerated, and is cost effective when compared to other thermal ablative techniques like cryoablation and microwave ablation.

Despite the considerable progress that has been made till date, a number of challenges remain. The technique definitely needs some refinements to address certain issues, which include definition of optimal methods and techniques for ablating tumors that can increase the volume of tissue destroyed at a single treatment session. RFA plays a useful role because of its many advantages; it is minimally invasive, it improves the survival and quality of life of patients with unresectable tumors, it reduces the cost and duration of hospital stay as compared to surgery, and it is associated with minimal complications and mortality.
